# Protein kinase D isoforms are dispensable for integrin-mediated lymphocyte adhesion and homing to lymphoid tissues

**DOI:** 10.1002/eji.201142004

**Published:** 2012-02-06

**Authors:** Sharon A Matthews, Hwee San Lek, Vicky L Morrison, Matthew G Mackenzie, Marouan Zarrouk, Doreen Cantrell, Susanna C Fagerholm

**Affiliations:** 1 Medical Research Institute, Ninewells Hospital and Medical School University of DundeeDundee, UK; 2 College of Life Sciences, University of DundeeDundee, UK

**Keywords:** Adhesion, Integrin, Lymphocyte, PKC, Protein kinase D (PKD)

## Abstract

Leukocyte function-associated antigen-1 (LFA-1) and very late antigen-4 (VLA-4) integrins are essential for lymphocyte adhesion, trafficking and effector functions. Protein kinase D (PKD) has previously been implicated in lymphocyte integrin regulation through regulation of Rap1 activity. However, the true role of PKD in integrin regulation in primary lymphocytes has not previously been investigated. The major PKD isoform in lymphocytes is PKD2. Here we employed PKD2-deficient mice, a specific PKD kinase inhibitor, as well as PKD-null DT40 B cells to investigate the role of PKD in integrin regulation in lymphocytes. We report that PKD2-deficient lymphocytes bound normally to integrin ligands in static and shear flow adhesion assays. They also homed normally to lymphoid organs after adoptive transfer into wild-type mice. DT40 B cells devoid of any PKD isoforms and primary lymphocytes pretreated with a specific PKD inhibitor bound normally to integrin ligands, indicating that multiple PKD isoforms do not redundantly regulate lymphocyte integrins. In addition, PKD2-deficient lymphocytes, as well as DT40 cells devoid of any PKD isoforms, could activate Rap1 in response to B-cell receptor ligation or phorbol ester treatment. Together, these results show that the PKD family does not play a critical role in lymphocyte integrin-mediated cell adhesion or lymphocyte trafficking in vivo.

## Introduction

Integrins are heterodimeric transmembrane proteins that are capable of bidirectional signalling across the plasma membrane. In resting lymphocytes these molecules are inactive. When the cell receives an activating stimulus, such as an antigen binding to T- or B-cell receptors or a chemokine binding to a chemokine receptor, specific intracellular signalling events are initiated which ultimately lead to the activation of integrins. This process is termed inside-out signalling [1–3]. The major integrins expressed by lymphocytes are leukocyte function-associated antigen-1 (LFA-1) (αLβ2, CD11a/CD18) and very late antigen-4 (VLA-4) (α4β1, CD49d/CD29). LFA-1 binds to intercellular adhesion molecules (ICAM-1/2) expressed on the surface of endothelial cells and other leukocytes, while VLA-4 binds to the extracellular matrix component fibronectin and to vascular cell adhesion molecule-1 (VCAM-1). After activation and subsequent binding to their ligands, these integrins are capable of transmitting signals into cells (termed outside-in signalling) that induce actin cytoskeleton reorganization and cell spreading events, which increase the overall avidity of cell–cell or cell–extracellular matrix adhesion.

In lymphocytes, integrin-mediated cell adhesion is critical for lymphocyte adhesion to endothelial cells under conditions of shear flow. Integrins are therefore essential for lymphocyte trafficking into secondary lymphoid tissues and sites of infection [4–7]. In addition, integrins are also important for optimal adhesion to antigen presenting cells, cell polarization and immunological synapse formation, and are therefore important for humoral and cell-mediated immune responses [8–15].

Integrin activation has been extensively studied in a variety of settings, and the small guanosine triphosphate (GTP) binding protein Rap1 has been identified as a central signalling component involved in integrin activation. Rap1 acts by recruiting downstream components such as RIAM (Rap1–GTP-interacting adapter molecule), which leads to recruitment of the integrin activator talin to the cytoplasmic tails of integrins, thereby initiating integrin activation [16]. Whether this sequence of events regulates lymphocyte integrins remains unclear, although Rap1 and talin have been identified as important regulators of integrin activation in lymphocytes [17–19]. In addition to talin, kindlin-3 has recently been identified as an essential regulator of integrin-mediated adhesion in lymphocytes [20–23].

In T cells, inside-out signalling to integrins can be initiated by T-cell receptor (TCR) ligation, which leads to activation of phospholipase C (PLCγ). Subsequent release of calcium and diacylglycerol activates protein kinase C (PKC) isoforms, as well as guanine exchange factors specific for Rap1, such as calcium and diacylglycerol-regulated guanine nucleotide exchange factor (CALDAG-GEF) [24–25] and RapGEF2 [26]. Another downstream target of PKC-signalling, namely protein kinase D (PKD), has also been implicated in regulating integrin-mediated cell adhesion in the Jurkat T lymphoma cell line. In this setting PKD was reported to mediate activation of Rap1 in response to TCR-triggering and phorbol ester treatment [27]. PKD has also been reported to control the recycling of αVβ3 and α5β1 integrins in fibroblasts [28–30] and to regulate the adhesion of renal carcinoma cells to endothelial cells [31]. In addition, PKD isoforms are also key regulators of the actin cytoskeleton and cell migration responses [32–36]. PKD has therefore been implicated in the regulation of cell adhesion and migration in a variety of settings. However, the true role of PKD isoforms in controlling integrin activation, lymphocyte adhesion and lymphocyte trafficking in primary cells in vivo is unclear at present. We therefore set out to investigate the relative importance of PKD enzymes in regulating integrin-mediated cell adhesion and migration responses in primary lymphocytes.

## Results

### PKD2-deficient lymphocytes display normal adhesion responses to ICAM-1 and fibronectin

**Figure 1:**
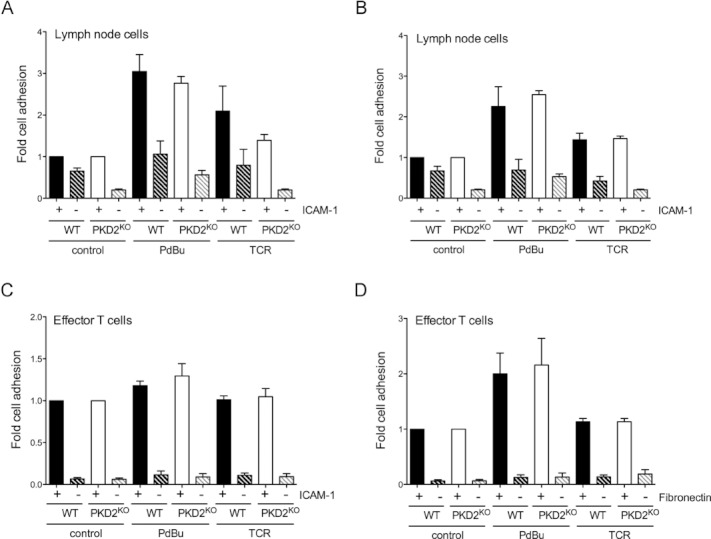
Adhesion of WT and PKD2-deficient lymphocytes to the integrin ligands ICAM-1 and fibronectin. (A and B) Lymphocytes isolated from the lymph nodes of wild-type (WT) and PKD2-deficient (PKD2 KO) mice were allowed to adhere to plastic surfaces coated (A) with or without 6 μg/mL ICAM-1 or (B) 1 μg/mL ICAM-1 for 20 min. Cells were left untreated or were treated with either 200n M PdBu or with 10 μg/mL of crosslinking TCR antibodies just prior to their addition to integrin-ligand coated plates. Data are shown as mean + standard error of the mean (SEM) of data pooled from four (A) or three (B) independent experiments, each performed in duplicate. (C/D) Adhesion of WT and PKD2 KO effector T cells to ICAM-1 (6 μg/mL, C) or fibronectin (10 μg/mL, D) coated surfaces was performed as in (A). Data are shown as mean + SEM of data pooled from five to eight independent experiments, each performed in duplicate or triplicate. There were no significant differences in the ability of WT versus PKD2-deficient cells to adhere to integrin ligands any of these assays (*p* > 0.05).

PKD2 is the major isoform expressed in primary naïve and effector murine lymphocytes, with the other family members either expressed at very low levels (PKD3) or not expressed at all (PKD1) ([37] and data not shown). We therefore initially focused on the role of PKD2 as a candidate regulator of integrin-mediated lymphocyte adhesion. Initial experiments confirmed normal expression of both the β1 and β2 integrin chains on the surface of PKD2-deficient lymphocytes (data not shown). We went on to analyse cell adhesion responses of both wild-type (WT) and PKD2-deficient lymphocytes to the β1 and β2 integrin ligands ICAM-1 and fibronectin using static adhesion assays. Adhesion of PKD2-deficient lymph node cells to either integrin ligand was comparable to that observed in WT lymphocytes, whether the cells were left untreated or whether they were stimulated with either phorbol esters or TCR ligands, to activate PKC, and therefore PKD, signalling (Fig. 1A and data not shown). Similarly, no defects were observed in integrin-mediated adhesion of untreated, phorbol ester-treated or antigen-receptor activated PKD2-deficient effector T cells (Fig. 1C and D), PKD2-deficient splenocytes or purified PKD2-deficient B-cells (data not shown). To exclude the possibility that maximal stimulatory conditions and/or excess integrin ligand amounts in the adhesion assays could overcome PKD2 deficiency in these cells, we also examined cell adhesion under different assay conditions. However, integrin-mediated adhesion of WT and PKD2-deficient lymphocytes to lower ligand densities or using shorter assay times did not reveal any observable adhesion defects in the PKD2-deficient lymphocytes compared to WT cells (Fig. 1B and data not shown). Thus, PKD2 does not play a significant role in integrin-mediated cell adhesion responses in primary lymphocytes under static conditions.

**Figure 2:**
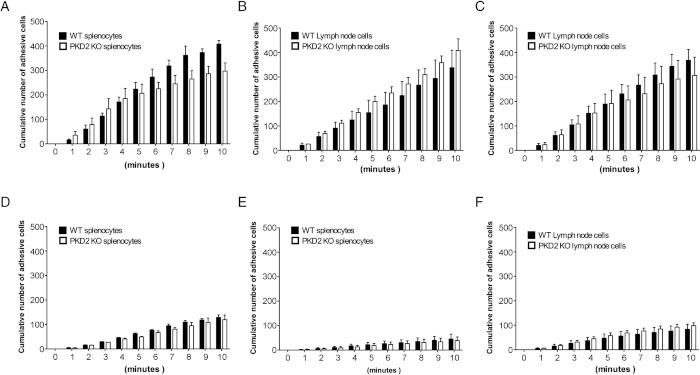
Integrin-mediated cell adhesion responses of WT and PKD2-deficient lymphocytes under conditions of fluid shear stress. Adhesion of WT (black bars) versus PKD2-deficient (PKD2 KO, white bars) spleen and lymph node lymphocytes to ICAM-1-coated surfaces under shear flow conditions was examined and quantified as described in the *Materials and Methods*. Surfaces were coated with either (D) 1 μg/mL or (A–C, E, F) 6 μg/mL ICAM-1. Lymphocytes were treated with either 10 μg/mL of crosslinking (A, D, E) BCR or (B, F) TCR antibodies or with (C) 200 nM PdBu before the start of the assay. Fluid shear flow rates were set at 0.3–0.5 dynes/cm^2^ (A–D) or 1 dynes/cm^2^ (E, F). Data are shown as mean + SEM of data pooled from three to four (A, C) or two (B, D, E, F) independent experiments, each performed in duplicate or triplicate. There were no significant differences in the ability of WT versus PKD2-deficient cells to adhere to integrin ligands in these assays (*p* > 0.05).

One of the most important functions of integrins is to mediate cell adhesion under shear flow conditions, a process that is critical to permit the firm adhesion of cells to high endothelial venules (HEV) [38]. Hence, we also investigated whether PKD2 was required to mediate high-affinity adhesion of primary lymphocytes to integrin ligands under conditions of fluid shear stress. WT lymphocytes isolated from secondary lymphoid tissues (spleen or lymph nodes) were able to activate their β2-integrins and adhere firmly to ICAM-1 under conditions of fluid shear stress in response to T- or B-cell antigen receptor stimulation or PdBu treatment (Fig. 2A to C). In contrast, resting WT lymphocytes were approximately threefold less efficient at binding to ICAM-1 under shear stress than activated WT lymphocytes (data not shown). Importantly, the ability of activated lymphocytes to adhere to the LFA-1 ligand ICAM-1 under conditions of fluid shear stress was not affected by PKD2-deficiency (Fig. 2A to C). Lowering the ligand density in the assay (Fig. 2D) or increasing the shear flow rate (Fig. 2E and F) did not reveal any adhesion defects in PKD2-deficient cells.

### PKD2 is not required for lymphocyte migration in vitro, nor for trafficking of lymphocytes in vivo

**Figure 3:**
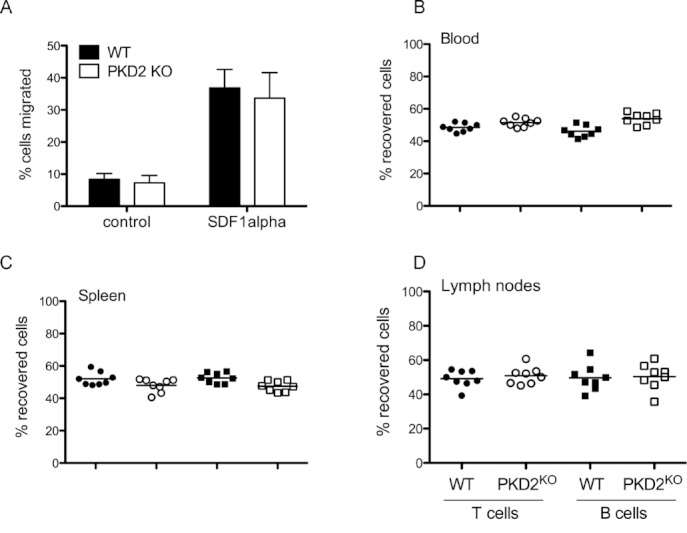
In vitro migration and in vivo trafficking of WT and PKD2-deficient lymphocytes to secondary lymphoid tissues. (A) Splenocytes from WT (black bars) and PKD2-deficient mice (PKD2 KO, white bars) were left to migrate across fibronectin-coated membranes for 4 h in response to the chemokine SDF-1α (250 ng/mL). Data are shown as mean ± SEM of data pooled from two independent experiments, each performed in duplicate. (B–D) Lymphocytes from WT mice labelled with CFSE and PKD2-deficient mice labelled with CellTrace Violet or CMTMR were mixed at a ratio of 1:1 before being injected into C57BL/6 host mice. Values indicate recovery of WT (black symbols) or PKD2-deficient (white symbols) TCRβ^+^ T cells (circles) or B220^+^ B cells (squares) as a percentage of the total recovered transferred cells from the (B) blood, (C) spleen and (D) lymph nodes 3 h after transfer. The data shown are pooled from two independent experiments, with each dot indicating one individual mouse (*n* = 8); horizontal bars indicate means.

Integrins are essential for the ability of lymphocytes to migrate from the bloodstream into secondary lymphoid tissues via the regulation of cell adhesion, spreading and migration. All three mammalian PKD isoforms have been identified as novel regulators of actin-driven cell migration in many cell types [33–36, 39–40]. As PKD1 is not expressed in naïve or effector lymphocytes and PKD3 is expressed only at very low levels ([37] and data not shown), we were interested in whether PKD2 was required to regulate migratory responses of primary lymphocytes. In an in vitro assay, WT or PKD2-deficient lymphocytes were added to transwell filters coated with the β1-integrin ligand fibronectin and left to migrate towards an stromal cell-derived factor 1 (SDF-1α) chemokine gradient for 4 h. As shown in Fig. 3A, fibronectin-mediated, SDF-1α-induced migration of PKD2-deficient lymphocytes was equivalent to that of WT lymphocytes. Similarly, there were no significant differences in the ability of WT and PKD2-deficient lymphocytes to migrate towards SDF-1α in vitro when the assay conditions were varied (shorter assay times, lower chemokine concentrations; data not shown). To confirm these results, we assessed the ability of PKD2-deficient lymphocytes to successfully migrate from the bloodstream into secondary lymphoid tissues in vivo by mixing WT and PKD2-deficient lymphocytes at a ratio 1:1 and injecting them into the tail vein of WT hosts. Subsequently, blood, lymph nodes and spleen were analysed for the presence of these transferred cells. As shown in Fig. 3B, PKD2-deficient and WT T and B cells were equally present in the blood, and importantly, PKD2-deficient lymphocytes could exit the blood and enter secondary lymphoid tissues normally.

**Figure 4:**
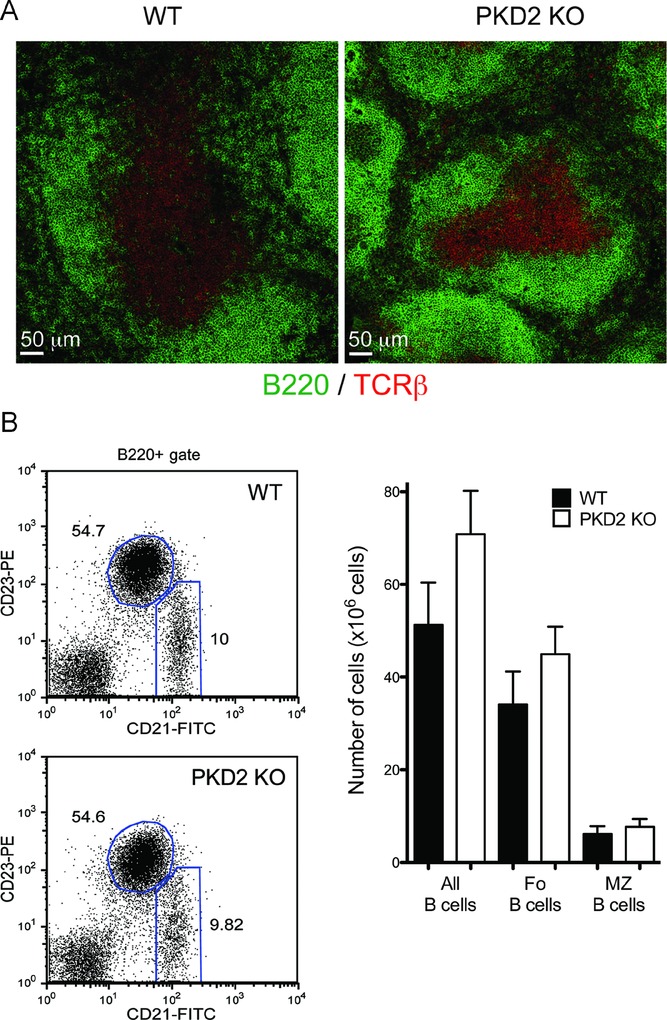
Analysis of lymphocyte compartmentalization within the spleens of WT and PKD2-deficient mice. (A) Spleen sections of WT (left) or PKD2-deficient (right) mice were stained with anti-B220 (green) and anti-TCRβ (red) antibodies. (B) B220^+^ MZ and follicular B-cell subsets in the spleens of 8–12-week-old WT (top) and PKD2-deficient (PKD2 KO, bottom) mice were analysed by flow cytometry using CD23 and CD21 antibodies, as described in the *Materials and Methods*. Graph represents the absolute cell numbers of the different B-cell subsets and data are shown as mean + SEM of nine mice, analysed in five separate experiments.

Another essential function for LFA-1 and VLA-4 integrins is to regulate lymphocyte entry into the splenic white pulp [7]. Absolute numbers of T and B lymphocytes in the spleens of PKD2-deficient mice are normal [37] and, as shown in Fig. 4A, splenic white pulp architecture in PKD2-deficient mice is also normal, with B220^+^ B and TCRβ^+^ T cells located within B-cell follicles and T cell zones, respectively. Integrins also play an important role in maintaining correct lymphocyte compartmentalization within the spleen, specifically by retaining marginal zone (MZ) B cells at the marginal zone [6]. We therefore asked whether MZ B-cell numbers and localization within the spleens of PKD2-deficient mice was defective or not. Histological analysis revealed normal marginal zone architecture in the spleens of PKD2-deficient versus WT mice (data not shown). Furthermore, the frequency and absolute numbers of IgM^Hi^CD21^Hi^CD23^Lo^ MZ B cells and IgD^+^CD21^Lo^CD23^Hi^ follicular B cells in the spleens of PKD2-deficient mice were comparable to that of WT mice (Fig. 4B). Collectively, these data argue that PKD2 does not play an important role in mediating adhesion and migratory responses of lymphocytes into, and positioning within, secondary lymphoid tissues in vivo.

A B-cell line devoid of any PKD isoforms can mediate normal adhesion to integrin ligands and a specific PKD inhibitor that abolishes PKD activity in cells has no effect on integrin-mediated adhesion in murine lymphocytes.

**Figure 5:**
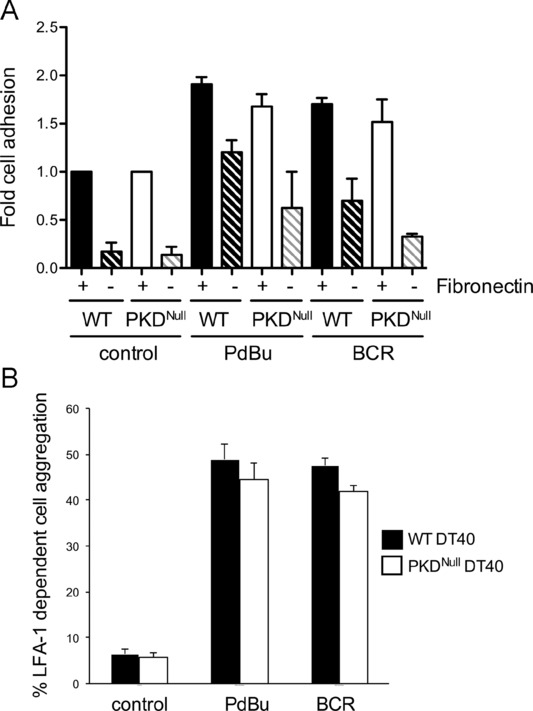
Analysis of integrin-mediated cell adhesion responses of WT and PKD-null DT40 B-cells. (A) Adhesion of WT versus PKD-null DT40 B cells to fibronectin-coated surfaces was examined as described in the *Materials and Methods*. Cells were left unstimulated or were treated with either 200 nM PdBu or were stimulated with a BCR ligand (10 μg/mL M4 mAb) just prior to their addition to fibronectin-coated plates. Data are shown as mean ± SEM of data pooled from four (control, PdBu) and two (BCR) independent experiments, each performed in duplicate. (B) LFA-1 mediated cell:cell adhesion of WT (black bars) and PKD-null (white bars) DT40 B cells was assessed as described in the *Materials and Methods*. Data are shown as mean + SEM of data pooled from three independent experiments, each performed in duplicate.

Functional redundancy between different PKD family members has been described previously [41]. Given that a second PKD isoform, PKD3, is also expressed in murine lymphocytes, albeit at very low levels [37], we wanted to address whether PKD isoforms could redundantly regulate integrin activity/function in lymphocytes. Therefore, we made use of a previously described avian PKD-null DT40 B-cell line that expresses no PKD isoforms at all. As shown in Fig. 5A, the ability of PKD-null DT40 B cells to adhere to the β1-integrin ligand fibronectin was not significantly different to that of WT DT40 B cells, either under resting conditions or after stimulation with either phorbol ester or a B-cell receptor (BCR) ligand. As avian ICAM-1 is not available and DT40 cells do not adhere to human or mouse ICAM-1, we turned to an aggregation assay to assess LFA-1 function in these cells. The LFA-1 ligand ICAM-1 is expressed on the surface of lymphocytes and LFA-1 can mediate cell–cell adhesion by binding to this ligand in-trans in response to specific extracellular stimuli [42–44]. We observed that PKD-null DT40 B cells could aggregate normally in response to both a BCR ligand or phorbol ester treatment (Fig. 5B), suggesting that LFA-1-mediated cell adhesion responses are normal in PKD-null DT40 B cells.

**Figure 6:**
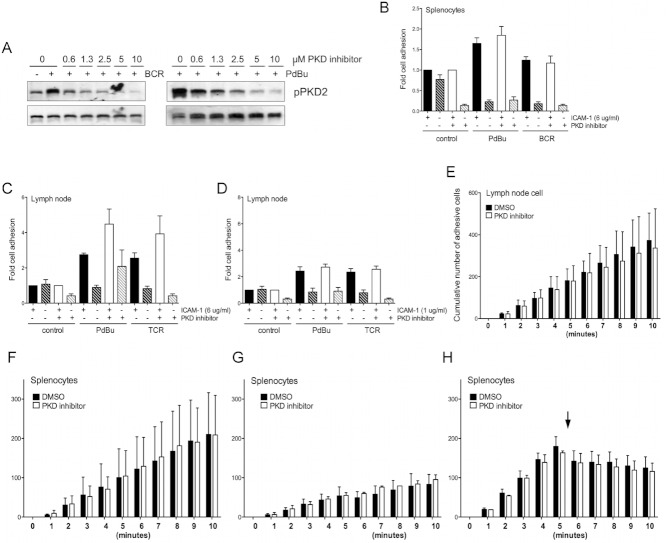
PKD isoforms do not redundantly regulate adhesion of murine lymphocytes to integrin ligands. (A) Primary murine splenocytes were pre-treated with dimethylsulfoxide (DMSO) solvent or with increasing concentrations of a PKD specific inhibitor (0.625–10 μM) for 1 h before stimulation with either a BCR ligand (10 μg/mL, left) or with 200 nM PdBu (right) for 30 min. The cells were then lysed and protein extracts analysed by SDS-PAGE and western blotting for auto-phosphorylated PKD2 and total PKD2 expression. Data are representative of two independent experiments. (B–D) Static adhesion assays were performed using lymphocytes isolated from the (B) spleens or (C, D) lymph nodes of WT mice. The cells were pre-treated with DMSO solvent or with 5 μM of a PKD specific inhibitor for 1 h before they were left untreated or were activated with either 200 nM PdBu or with 10 μg/mL of crosslinking BCR (splenocytes) or TCR (lymph node cells) antibodies just prior to their addition to plates coated with 6 μg/mL ICAM-1 (B,C) or 1 μg/ml ICAM-1 (D). Data are shown as mean + SEM of data pooled from four (splenocytes) or three (lymph node cells) independent experiments, each performed in duplicate. (E–G) Adhesion of antigen receptor activated control (DMSO, black bars) or PKD-inhibitor treated (5 μM, white bars) WT spleen and lymph node cells to surfaces coated with 6 μg/mL ICAM-1 under low (0.3–0.5 dynes/cm^2^; E, F) or high (1 dynes/cm^2^; G) shear flow conditions was analysed as described above. (H) Antigen receptor activated control (DMSO, black bars) or PKD-inhibitor treated (5 μM, white bars) splenocytes were allowed to adhere to ICAM1 (6 μg/mL) coated surfaces under low (0.3–0.5 dynes/cm^2^) shear flow conditions for 5 min before switching to high shear flow (1 dyne/cm^2^; arrow). Data (E–H) are shown as mean + SEM of data pooled from two independent experiments, each performed in duplicate. There was no significant effect of the PKD inhibitor on the adhesion of WT lymphocytes to adhere to integrin ligands in any of these assays (*p* > 0.05).

To ensure that the normal adhesion properties of PKD-null cells were not an artefact due to changes in signalling pathways in the immortalized DT40 cell line, we also performed experiments with a recently described PKD inhibitor that inhibits all three mammalian PKD isoforms with a high degree of selectivity [45–46]. Pre-treatment of WT murine splenocytes with 5–10 μM of this inhibitor abolished PKD2 activity, as assessed by western blotting with a phosphospecific antibody that detects active, autophosphorylated PKD1/2 (Fig. 6A) [47]. In contrast, adhesion of lymph node cells and splenocytes to ICAM-1 and fibro-nectin under basal and stimulated conditions were similar for both non-treated and PKD inhibitor-treated cells (Fig. 6B and C and data not shown). Varying ligand concentrations, amounts of stimulatory agents or incubation time for the adhesion assays also did not reveal any adhesion defects in the adhesion properties of the PKD-inhibitor treated cells (Fig. 6D and data not shown). Similarly, the adhesion properties of PKD inhibitor-treated lymphocytes to low or high ICAM-1 ligand densities under conditions of increasing shear flow (0.3–1 dynes/cm^2^) was comparable to that of WT cells (Fig. 6E to G and data not shown). We also performed detachment assays to assess whether there were any significant differences in the strength of adhesion of PKD inhibitor-treated lymphocytes for ICAM-1. Here, non-treated and PKD inhibitor treated lymphocytes were stimulated with a BCR ligand and then allowed to adhere to ICAM-1 ligands at low shear flow rate (0.3–0.5 dynes/cm^2^) before the flow rate was increased to 1 dynes/cm^2^. Under these conditions we observed no significant differences in the rate of detachment of PKD2 inhibitor treated cells versus control cells from ICAM-1 coated surfaces (Fig. 6H). Based upon these experiments, we conclude that PKD isoforms do not play an important role in regulating integrin-mediated cell adhesion responses in lymphocytes.

### PKD deficiency does not affect Rap1 activation in lymphocytes

**Figure 7:**
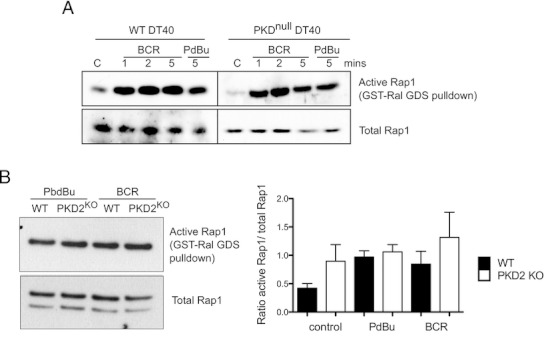
Normal Rap1 activation in PKD-deficient lymphocytes. (A) WT and PKD-null DT40 B cells were left untreated (control, C) or were stimulated with a crosslinking BCR antibody for 1–5 min or with 200 nM PdBu for 5 min before Rap1 activity was assessed, as described in the *Materials and Methods*. Total cell lysates and samples that had been enriched for active GTP-loaded Rap1 were immunoblotted with a Rap1 polyclonal antibody. Data are representative of two independent experiments. (B) Rap1 activity in control, phorbol ester (PdBu) or antigen receptor (BCR) activated WT and PKD2-deficient primary murine splenocytes was analysed as described in the *Materials and Methods*. Left, western blot analysis of total Rap1 in whole cell lysates (4% of input) and active Rap1 in samples been enriched for GTP-loaded Rap1. Right, western blot data from five independent experiments were quantified using ImageJ64 software and is presented as the ratio of active Rap1/total Rap1.

PKD has previously been described to regulate the activation of the integrin regulator Rap1 in the Jurkat T-cell line [27]. Therefore, we analysed the ability of PKD-null DT40 B cells that lack expression of all PKD isoforms to activate Rap1 by utilizing a Rap1 pull-down assay. We observed that both BCR ligation and phorbol ester treatment could induce activation of the key integrin regulator Rap1 in both WT and in PKD-null DT40 cells (Fig. 7A). Furthermore, Rap1 activity in PKD2-deficient murine lymphocytes was comparable to that of WT lymphocytes (Fig. 7B) and was not influenced by pretreatment with a PKD specific inhibitor (data not shown). Taken together, these results suggest that inside-out signalling to integrins is normal in PKD2-deficient lymphocytes and in PKD-null DT40 B cells, indicating that the PKD family per se does not play a significant role in regulating lymphocyte integrin function.

## Discussion

The PKC and PKD families of protein kinases have previously been implicated in regulation of lymphocyte integrins; however, the role of PKD in these events has not previously been clarified in primary lymphocytes. Therefore, we set out to investigate the role of PKD in integrin regulation in these cells. Surprisingly, deletion of the major PKD isoform in lymphocytes, PKD2 [37], did not lead to a deficiency in lymphocyte binding to integrin ligands in adhesion assays conducted without or with shear flow, indicating that PKD2 does not play a critical role in integrin regulation in lymphocytes. In addition, we found no evidence of integrin defects in PKD2-deficient mice by investigating the homing responses of PKD2-deficient lymphocytes and also the localization of PKD2-deficient cells within lymphoid tissues. By using DT40 avian B cells devoid of all PKD isoforms, as well as a specific PKD inhibitor, we excluded the possibility that the functional integrin phenotype in these cells was due to redundant expression of another PKD isoform [37].

Rap1 is a critical component of the inside-out signalling pathway to integrins in lymphocytes, and PKD has previously been implicated in Rap1 activation in Jurkat T cells using siRNA-approaches [27]. Therefore, we investigated Rap1 activation in DT40 B cells devoid of all PKD isoforms and in primary murine lymphocytes lacking PKD2 (the major PKD isoform expressed in these cells). In contrast to previous experimental data [27], Rap1 activation in these cells in response to BCR ligation or phorbol ester stimulation appeared to be normal, confirming that PKD does not play a role in Rap1 regulation or inside-out signalling to lymphocyte integrins. The difference between our data and that of Mederios et al. [27] presumably reflects differences in the regulation of Rap1 activity in primary lymphocytes versus a transformed Jurkat T-cell line. Taken together, our results confirm that in primary lymphocytes, PKD isoforms do not play a role in integrin activation, cell adhesion or lymphocyte trafficking events. In the future it will be interesting to determine whether PKD enzymes have a role in regulating lymphocyte adhesion/migration responses during an immune ‘challenge’ in vivo.

PKC isoforms, which are important upstream regulators of PKD in lymphocytes [48–49], have a crucial role in regulating integrin-mediated adhesion in primary lymphocytes, at least in T cells [26, 50]. Thus, in T cells the PKCθ isoform regulates the activation of RapGEF2, and therefore Rap1 activation [26]. In agreement with these findings, we observed that low micromolar concentrations of Ro-31-8220 (a general PKC inhibitor) and Go6976 (which inhibits classical PKCs) block the ability of primary murine B cells adhere to integrin ligands (data not shown), suggesting that PKC enzymes also regulate integrin activity/function in B cells. PKCβ is a central PKC isoform in B cells, and previous studies of knockout mice have revealed that PKCβ plays a central role in regulating B-cell survival and humoral immune responses [51–53]. However, WT murine B cells pre-treated with a PKCβ specific inhibitor, enzastaurin, adhere normally to integrin ligands (data not shown). Thus, it remains unclear exactly which PKC enzyme(s) regulate integrin-mediated cell adhesion responses in primary B cells.

In conclusion, we report that PKC, but not PKD, enzymes are important for regulating integrin activity and function in primary T and B lymphocytes. Similarly, it has recently been reported that PKD2 does not mediate PKC-dependent signalling to the αIIbβ3 integrin in platelets [54], confirming that the PKD family is not important for integrin regulation in a number of different systems where integrin activation plays crucial biological roles.

## Materials and methods

### Reagents and antibodies

Phorbol 12,13-dibutyrate (PdBu) was obtained from Sigma-Aldrich. The PKD inhibitor described previously [45–46] was synthesized by the DSTT unit, College of Life Sciences, The University of Dundee. The integrin ligands fibronectin and ICAM-1 were from Calbiochem and R&D Systems, respectively. F(ab)’2 fragments of anti-mouse IgMμ antibodies used to stimulate the B-cell antigen receptor complex were from Jackson Immunoresearch. The anti-CD3 monoclonal antibody (clone 2c11) used to stimulate the T-cell antigen receptor was from R&D Systems. The M4 monoclonal chicken anti-IgM hybridoma cell line was obtained from Riken (Japan) and was concentrated from serum-free hybridoma supernatants by ammonium sulphate precipitation.

### Cell culture

PKD-null (PKD1/3 double knockout) DT40 B cells have been described previously [41]. Lymphocytes from PKD2-deficient mice were isolated from spleen and lymph node tissues as previously described [37]. Naïve B cells were purified from mouse spleen using CD43 Dynabeads (Invitrogen) according to the manufacturer's instructions. To generate effector T cells, splenocytes were activated for 2 days with 0.5 μg/mL anti-CD3 antibody (2C11). Cells were then washed free of activating agent and thereafter maintained in 20 ng/mL IL-2 and used for experimental purposes 4–5 days later. All mice were bred and maintained under specific pathogen-free conditions in the Wellcome Trust Biocentre at the University of Dundee in compliance with UK Home Office Animals (Scientific Procedures) Act 1986 guidelines.

### Static cell adhesion and aggregation assays

Fibronectin or ICAM-1 were coated in duplicate/triplicate onto flat-bottom 96-well Maxisorp plates (Nunc) by overnight incubation at 4°C. The wells were blocked with 1% milk/phosphate-buffered saline (PBS) for 1 h at 37°C. The cells were suspended in adhesion medium (RPMI with 0.1% bovine serum albumin (BSA), 40mM Hepes, 2mM MgCl_2_) before being added to blocked wells. The cells were allowed to adhere for 20 or 30 min at 37°C before unbound cells were removed by gentle washing in PBS/2 mM Mg^2+^. The bound cells were lysed in 1% Triton-X-100, 50 mM sodium acetate, pH 5.0 containing 3 mg/mL p-nitrophenylphosphate (Calbiochem) and incubated for 1 h at 37°C in the dark. The reaction was terminated with 1M NaOH and the absorbance at 405 nm was measured. Aggregation assays were performed as described previously [55].

### Shear flow cell adhesion assays

Cells at a density of 1×10^6^ cells/mL were pre-incubated with 200 nM PdBu or with 10 μg/mL of stimulatory anti-BCR or anti-TCR antibodies for 5 min in adhesion medium (RPMI with 0.1% BSA, 40 mM Hepes and 2 mM MgCl_2_) at 37°C. The cell suspension was then injected into a flow system that used a silicone tubing loop connected to a Multi-phaser NE-1000 syringe pump (New Era Pump Systems Inc.), allowing the cells to flow over ICAM-1-coated VI 0.4 Ibidi μ slides at a low (0.3–0.5 dynes/cm^2^) or high (1 dynes/cm^2^) continuous shear flow-rate over a 10-min period. Cells were monitored by microscopy and the number of adherent cells in the field of view at each timepoint was determined at 1 min intervals by manual counting.

### Transwell chemotaxis assays

Transwell membranes (5 μm; Corning Costar) were coated overnight at 4°C with 10 μg/mL fibronectin and then blocked with 2% BSA/PBS for 1 h at 37°C. Lymphocytes (1×10^6^ in 100 μL assay medium, RPMI/0.5% BSA) were added to the top of the Transwell and 600μL of assay buffer containing 250 ng/mL SDF-1α/CXCL12 chemokine (C-X-C motif) ligand 12 was added to the bottom well. Cells were left to migrate for 4 h at 37°C. Cell migration to the bottom well was quantitated using fluorescence-activated cell sorter (FACS) calibrite beads (BD Biosciences) on a FACS Calibur flow cytometer and analysed using FlowJo software (Treestar) or by manual cell counting.

### In vivo lymphocyte homing assays

Lymphocytes isolated from WT and PKD2-deficient mice were labelled with carboxyfluorescein succinimidyl ester (CFSE) or CellTrace Violet/CMTMR dyes (Molecular Probes, Invitrogen), respectively; washed and mixed in a 1:1 ratio in sterile PBS. Cell suspensions (10^7^ cells) were injected into the tail vein of C57BL/6 host mice, and 3 h later the mice were killed and tissues taken for quantification of CFSE and CellTrace Violet-labelled T and B cells by flow cytometry. Values indicate the recovery of injected WT or PKD2-deficient T and B lymphocytes as a percentage of the total recovered transferred cells isolated from blood, spleen and lymph nodes.

### Rap1 pull-down assay

Rap1 activity was determined using a Rap1 pull-down assay kit to isolate activated (GTP-loaded) Rap1 molecules (GST-RalGDS-RBD pull-down assay, Thermo Scientific). Prior to the pull-down assay, cells were suspended at a concentration of 2×10^7^/mL, washed with PBS, resuspended in RPMI/40 mM Hepes/0.1% BSA and rested for 15 min at 37°C. The cells were then stimulated with 200 nM PdBu or with 10 μg/mL of stimulatory anti-BCR antibodies for 5 min. Cell lysates were prepared and used in the pull-down assay according to the manufacturer's instructions. Total lysates (4% of input) and pull-down samples were analysed for Rap1 content using standard sodium dodecyl sulphate-polyacrylamide gel electrophoresis (SDS-PAGE) and immunoblotting techniques. Analysis of Rap1 activity in the DT40 cell lines was performed similarly, using GST-RalGDS-RBD produced ‘in-house’ and an anti-Rap1 antibody that recognizes avian Rap1 (Santa Cruz clone 121).

### Flow cytometry

Antibodies conjugated to FITC, PE, PerCP-Cy5.5 and allophycocyanin were obtained from either BD Biosciences or eBioscience. Fc receptors were blocked with mouse Fc block (CD16/CD32; clone 2.4G2) and cells were stained for surface expression of the following antigens (antibody clones are in parentheses): TCRβ (H57-597), B220 (RA3-6B2), CD21/35 (7G6), CD23 (B3B4), CD19 (ID3). Live cells were gated according to their forward scatter and side scatter. Data were acquired on an LSR Fortessa flow cytometer (Becton Dickinson) and analysed using FlowJo software (Treestar).

### Histology

Spleens harvested from WT and PKD2-deficient mice were frozen in isopentane and stored at −80°C. Cryosections (8–10 μM) were prepared, fixed in acetone and blocked with 1% BSA/PBS before they were stained with TCRβ-Alexa Fluor^633^ (red) and B220-Alexa Fluor^488^ (green). Images were acquired on a Zeiss LSM 710 Meta microscope equipped with ZEN 2009 software (version 5.5.0.375), an EC Plan-Neufluar 20×/0.5 M27 objective and 488 nm and 633 nm lasers with appropriate filters.

### Statistical analysis

Statistical analyses were performed using two-tailed unpaired Student's *t* test (or for flow adhesion data, two-way ANOVA), GraphPad Prism 5.0d for Macintosh (GraphPad Software). *p* values < 0.05 were considered to be statistically significant.

## Abbreviations

LFA-1: Leukocyte function-associated antigen-1
MZ: marginal zone
PKC: protein kinase C
PKD: protein kinase D
VLA-4: very late antigen-4
